# No association between genetic variants in *MAOA*, *OXTR*, and *AVPR1a* and cooperative strategies

**DOI:** 10.1371/journal.pone.0244189

**Published:** 2020-12-23

**Authors:** María I. Rivera-Hechem, Carlos Rodríguez-Sickert, Ricardo A. Guzmán, Tadeo Ramírez-Parada, Felipe Benavides, Víctor Landaeta-Torres, Mauricio Aspé-Sánchez, Gabriela M. Repetto

**Affiliations:** 1 Centro de Investigación en Complejidad Social (CICS), Facultad de Gobierno, Universidad del Desarrollo, Santiago, Chile; 2 Centro de Genética y Genómica, Facultad de Medicina, Clínica Alemana Universidad del Desarrollo, Santiago, Chile; Middlesex University, UNITED KINGDOM

## Abstract

The effort to understand the genetic basis of human sociality has been encouraged by the diversity and heritability of social traits like cooperation. This task has remained elusive largely because most studies of sociality and genetics use sample sizes that are often unable to detect the small effects that single genes may have on complex social behaviors. The lack of robust findings could also be a consequence of a poor characterization of social phenotypes. Here, we explore the latter possibility by testing whether refining measures of cooperative phenotypes can increase the replication of previously reported associations between genetic variants and cooperation in small samples. Unlike most previous studies of sociality and genetics, we characterize cooperative phenotypes based on strategies rather than actions. Measuring strategies help differentiate between similar actions with different underlaying social motivations while controlling for expectations and learning. In an admixed Latino sample (n = 188), we tested whether cooperative strategies were associated with three genetic variants thought to influence sociality in humans—*MAOA*-uVNTR, *OXTR* rs53576, and *AVPR1* RS3. We found no association between cooperative strategies and any of the candidate genetic variants. Since we were unable to replicate previous observations our results suggest that refining measurements of cooperative phenotypes as strategies is not enough to overcome the inherent statistical power problem of candidate gene studies.

## Introduction

The questions of why and when people are willing to cooperate, bearing individual costs in the pursuit of collective benefit, have been a major focus in the social and natural sciences [1–4]. Although cooperation is widespread among humans, there is considerable diversity among cooperative behaviors [5–8]. Evidence supporting the heritability of social traits has encouraged efforts to understand the genetics underlying this diversity [9–11]. Although several studies have searched for genetic variation associated with cooperation and related behaviors, such as trust, reciprocity, and altruism, this task has remained elusive [3].

The existing literature relies heavily on studies that test the association between social phenotypes and a handful of candidate gene variants using small sample sizes of few hundred individuals (i.e. candidate genes studies) [12, 13]. The results of these studies are inconsistent and usually not replicable [14–17]. The most accepted explanation for the lack of robust findings is that most candidate gene studies lack statistical power given the small effects that single variants may have on complex social traits [14, 18–20]. The lack of robust associations could also be a consequence of a poor characterization of cooperative phenotypes. This possibility, which we aim to address in this study, has largely been overlooked in the literature.

A common approach to characterizing social phenotypes is to measure actions displayed in incentive-based tasks grounded on game-theoretic experimental paradigms (see Table 1 for a summary of this literature). However, actions displayed in these tasks result from the interaction of multiple cognitive processes. For example, in tasks for which the outcome is given by the simultaneous decisions of multiple players, actions are influenced by the expectations of subjects about the behavior of others [21]. Additionally, in tasks involving repeated decisions, actions are influenced by learning [22]. Expectations and learning are likely to involve different neural networks and structures [23]. Therefore, unpacking cooperative traits into more elementary constructs could help elucidate associations with particular genetic variants [24].

**Table 1:** Summary of previous studies of genetics and sociality using incentive-based tasks.

Reference	Sample size	Population	Incentive-based task	Involves expectations (i.e. simultaneous decisions)	Involves learning (i.e. repeated decisions)	Social trait	Candidate gene	Result
Knafao et al., 2008 [37]	203 (101 women)	College students from Israel	Dictator game	No	No	Altruism	*AVPR1A*	Carriers pf shorter variants of the *AVPR1a* RS3 repeat showed less altruistic behavior than carriers of longer variants.
Israel et al., 2009 (first sample) [75]	203 (102 women)	College students from Israel and their families	Dictator game	No	No	Altruism	*OXTR*	Variants of rs1042778, rs237887, and rs2268490 were association with altruism.
Israel et al., 2009 (second sample) [75]	98 (all women)	Mothers from Israel	Dictator game	No	No	Altruism	*OXTR*	Variants of rs1042778, rs237887, and rs2268490 were association with altruism.
Apicella et al., 2010 [67]	684 (80% women)	Swedish twins	Dictator game	No	No	Altruism	*OXTR*	No association
			Trust game with trustee role in strategy method	Yes, for the trustor	No	Trust and trustworthiness		
Zhong et al., 2010 [76]	208 (54% women)	Chinese Han	Ultimatum game	Yes	No	Fairness	*DRD4*	DRD4 is associated with fairness preference
Avinun et al., 2011 [43]	158 (81 women)	Israeli preschool twins	Dictator game	No	No	Altruism	*AVPR1A*	Carriers of the variant with 327 bp showed lower altruism compared to other subjects
Mertins et al., 2011 [29]	96 (60 women)	Students at the University of Trier in Germany	Repeated public good game	Yes	Yes	Cooperation	*MAOA*	Men carriers of the low activity alleles cooperate significantly less than those carrying the high activity alleles
Krueger et al., 2012 [66]	108 (all men)	College students with European ancestry	Trust game	Yes	Yes	Trust and trustworthiness	*OXTR*	GG genotype for rs53576 showed higher trust than AA and AG genotypes
Chew et al., 2013 (first sample) [77]	208 (112 women)	Han Chinese	Ultimatum game	Yes	No	Fairness	*AR*, *ERα*, and *ERβ*	*AR* associated with minimal acceptable offers in men. *Erβ* associated with minimal acceptable offers in women.
Chew et al., 2013 (second sample) [77]	257 (125 women)	Israeli	Ultimatum game	Yes	No	Fairness	*AR*, *ERα*, and *ERβ*	*Erβ* marginally associated with minimal acceptable offers in women.
Mertins et al., 2013 [27]	91 (58 women)	Students at the University of Trier in Germany	Public good game using strategy method	No	No	Cooperation	*MAOA*	Women carriers of the low activity alleles were less likely to behave as weak free riders compared to women carrying high activity alleles
Reuter et al., 2013 [78]	130 (105 women)	Caucasians	Ultimatum game	Yes	No	Fairness	*DRD2* and *DRD4*	4/4 genotype for the *DRD4* variant showed a higher minimal acceptable offer than other genotypes
Schroeder et al., 2013 [79]	184 (107 women)	Students at Newcastle University in England	Repeated Public good game	Yes	Yes	Cooperation	*SLC6A4* and *HTR2A*	*SLC6A4* was associated with cooperation in the absence of punishment. In the presence of punishment, cooperation was associated with *HTR2A*
Feng et al., 2015 [36]	204 (100 women)	Students at the Emory University in the United States of America	Iterated sequential Prisoner’s Dilemma Game	No	Yes	Cooperation	*OXTR*	Sex differences in effects of intranasal oxytocin treatment for individuals with the GG genotype
Nishina et al., 2015 [35]	470 (242 women)	Non-student Japanese	Trust game with trustee role in strategy method	Yes, for the trustor	No	Trust and trustworthiness	*OXTR*	GG genotype for *OXTR* rs53576 showed higher trust than AA genotype in men
Wang et al., 2016 [38]	278 (150 women)	Chinese Han	Dictator game	No	No	Altruism	*AVPR1A*	Men with relatively short variants of RS3 allocated less money to others compared with men carrying two copies of long variants
Nishina et al., 2019 [39]	434 (221 women)	Non-student Japanese	Trust game with trustee role in strategy method	Yes, for the trustor	No	Trust and trustworthiness	*AVPR1a*	Men with a short form of *AVPR1a* showed more trust than those carrying other variants. Additionally, subjects with a short form of *AVPR1a* displayed higher trustworthiness

Summary of the design features of candidate gene association studies using incentive-based tasks to measure social traits.

Strategies are game-theoretic constructs that can reflect social motivations while controlling for expectations and learning. A strategy is a player’s contingent plan specifying her/his actions in response to all the possible actions of the other players. The standard prediction in economics is that, in contexts that require cooperation, individuals will choose to free-ride no matter what others do. However, strategies in cooperative contexts are diverse and the “free-riding” strategy is not the most prevalent [5, 6, 8]. Most people condition their cooperation on their counterparts’ behaviors. The majority chooses to closely match the levels of cooperation of their counterparts, a strategy that is usually referred to as “conditional cooperation”[2]. Some individuals prefer to match the levels of cooperation of their counterparts only up to a certain level at which they start decreasing their contributions, a strategy that is referred to as “hump-shaped” [5]. In situations where the actions of others are unknown, those that like to condition their actions on what others do will behave based on their expectations about the decisions of others [2]. Therefore, if only actions are observed, as is the case in most candidate gene studies, it is not possible to discriminate between conditional cooperation, hump-shaped, and free-riding strategies. This differentiation is possible, however, if incentive-based tasks are designed to elicit strategies rather than actions. Specifically, to use the “strategy-method” [25] rather than the standard direct response method allows identifying underlying strategies in the context of a public goods game (PGG), a task widely used to study cooperation [5].

Recent evidence supports the inheritability of cooperative strategies [11] and sheds light on its neurological basis [26]. To our knowledge, only Mertins et al. [27] have reported association of a genetic variant with cooperative strategies, *MAOA*-uVNTR, located in the gene that codes for monoamine oxidase A, which metabolizes monoamine neurotransmitters. Variants in this gene lead to a lower expression (MAOA-L) or higher expression (MAOA-H) of monoamine oxidase A [28]. Mertins et al. [27] found that women with MAOA-L variants are less likely to behave like free-riders than MAOA-H carriers. Similarly, it has been observed that women carrying MAOA-L variants cooperate more in repeated interactions [29] and that MAOA-L genotypes correlate with social sensitivity [30].

Other variants that have been associated with social behaviors are in genes that encode receptors for oxytocin and vasopressin, two neurotransmitters highly linked to sociality [31]. The single nucleotide variant rs53576 in *OXTR* and the microsatellite RS3 in *AVPR1a* have emerged as promising candidates for social behavior. Individuals homozygous for the G allele (GG) of *OXTR* rs53576 show higher levels of empathy [32], sociality [33, 34], and higher levels of trust [35] compared to individuals with one or two copies of the A allele (AA/AG). It has also been suggested that the G allele modulates the effect of oxytocin in cooperative interactions [36]. The lengths of variants of *AVPR1a* RS3 also correlate with cooperation-related social traits. For instance, individuals with relatively long repeats in *AVPR1a* RS3 are more altruistic [37, 38], but are less trusting and disposed to reciprocity [39].

We aim to explore whether more refined measures of cooperative phenotypes—underlying strategies rather than observable actions—support the replicability and robustness of previously reported associations. We replicate the analysis of Mertins et al. [27], by testing the association between cooperative strategies and *MAOA-*uVNTR variants in an admixed Latino population and extended it by including *OXTR* rs53576, and *AVPR1a* RS3 as additional candidates (n = 188).

## Methods

### Subjects and recruitment

Our sample consisted of 200 Chilean students (18 to 25 years old, women = 109) from Universidad del Desarrollo (UDD), in Santiago, Chile. Subjects were recruited two weeks prior to the experimental sessions by emailing all students an invitation to participate in a study about genetics and decision-making and recruitment posters were distributed on campus. Volunteers filled out an online form with their contact information and availability. The only inclusion criterion was that subjects be students at UDD at the time of participation.

### Experimental procedures

We conducted 10 sessions in a computer laboratory at UDD between June and September 2013. Subjects were notified of an experimental session via email and were offered a show-up fee of $2.500 CLP, plus additional earnings from the incentive-based task. In each session, 20 students entered the room and were seated in front of an individual computer. The facilitator informed the subjects that their participation would consist of playing a four-person PGG, after which they would provide a saliva sample. Subjects were also informed that decisions would be recorded anonymously and that they could leave the experiment at any moment. The game was programmed in z-Tree [40] and communication during the game was not allowed. A printed copy of the instructions of the game was handed to each participant and the facilitator read them aloud at the beginning of the session (S1 Text). Examples of outcomes were shown, and questions were answered aloud before the game started. Our protocol was approved by the UDD Research Ethics Committee.

Each individual in the game was given 20 tokens (valued $250 CLP) and had to privately decide how many tokens to contribute to the public good and how many to keep for themselves. Contributions to the public goods were doubled and divided into equal parts among the four members in the group, regardless of how many each member contributed. The game’s payoff function was:

$$\pi_{i}=\left(20-g_{i}+0.5\sum_{j=1}^{j=4}g_{j}\right)\times \$250\;CLP$$

Where *π_i_* is the final payoff of subject *i*, *g_i_* ∈ {0,1,…, 20} is the contribution of individual *i* to the public goods, and *g_j_* ∈ {0,1,…, 20} is the contribution of each member of the group. As the marginal gain of contributing one token to the public good is 0.5 while the marginal gain of keeping it is one, we expected no contributions to the public good under the assumption of self-interested, profit-maximizing individuals.

We used the PGG game protocol developed by Fischbacher et al. [5] in which subjects are asked to make two types of decisions: an “uninformed contribution” and a “contingent contribution”. The uninformed contribution was the answer to the question: You have 20 tokens; how many tokens will you contribute to the public goods? (S1 Fig). This question did not provide subjects with information about what other members of the group were contributing. Consequently, this decision involved individual expectations about the contributions of others. The contingent contribution required that subjects answer the question of how many of their 20 tokens they would contribute to the public goods given a scenario in which the other members of the group contribute an average of ${\bar{g}}_{j\neq i}$ tokens (rounded to the integer), with ${\bar{g}}_{j\neq i}$ ∈ {0,1, 2…20} (S2 Fig). The answer to this question elicited cooperative strategies which exclude the confounding effects of intertemporal strategies, learning or expectations about the cooperative behavior of others.

After subjects provided their answers for their uninformed and contingent contributions, they were randomly and anonymously matched by the software into groups of four. The uninformed contributions of three random players in the group were averaged and rounded to the integer to obtain ${\bar{g}}_{j\neq i}$, which was then employed to find the contribution of the fourth player based on her/his contingent contribution. This provided the total contribution to the public goods, and individual payoffs were calculated. This procedure ensured that both answers were incentive compatible as both could be considered to calculate individual payoffs.

Saliva samples were collected at the end of the session using Saliva Self-Collection Kit OG 500 (DNA Genotek, Canada). Each subject provided a sample in a tube labeled with the same identification code under which the subject’s answers in the game were recorded. Subjects collected their profits privately in a separate room.

### Genotyping

DNA was successfully extracted from 188 samples (women = 107). The three candidate variants were analyzed as described in the protocols of previous studies [41–43]. Results revealed four alleles for *MAOA*-uVNTR in our sample presenting 3.5, 4.5, 5.5, and 6.5 repeats (S1 Table). These alleles correspond respectively to the 3, 4, 5, and 6 repeats alleles observed in previous studies [44]. Given the low frequencies of the 5.5 and 6.5 repeats alleles in our sample, we excluded their carriers from the analysis. Since the *MAOA* gene is in the X chromosome, men only have one allele for *MAOA*-uVNTR, therefore genotypes for men are 4.5 and 3.5 repeats, equivalent to the MAOA-H and MAOA-L, respectively [28]. In the case of women, one of the two X chromosomes in somatic cells becomes transcriptionally inactive early in development [45]. We cannot determine which of the alleles is being expressed in women that are heterozygous for *MAOA*-uVNTR, therefore we excluded them from the analysis. This left us with the two homozygous genotypes of *MAOA-*uVNTR in women—4.5/4.5 and 3.5/3.5 repeats—equivalent to the MAOA-H and MAOA-L variants, respectively [28]. Consequently, genotypes for *MAOA* u-VNTR were coded under “MAOA-H” or “MAOA-L” in both women and men.

Genotypes for *OXTR* rs53576 were coded as “GG”, “GA”, and “AA”. Alleles for *AVPR1a* RS3 were classified as “Short” if they were between 324 bp to 341 bp long and as “Long” if they were between 342 bp to 356 bp long (S2 Table). This cutoff was established to ensure that both groups were balanced in the number of observations. This classification method is often used for microsatellite repeats due to a usually high number of low‐frequency alleles [37]. Genotypes for the RS3 *AVPR1a* were coded as “Short/Short”, “Short/Long” and “Long/Long”.

The resulting genotype distribution satisfies Hardy-Weinberg equilibrium for *AVPR1a* RS3 (X^2^ = 2.84, p = 0.09) and *MAOA* u-VNTR (X^2^ = 0.18, p = 0.67, tested only for women because it is a sex-linked variant), but not for *OXTR* rs53576 (X^2^ = 5.29, p = 0.02) (S3 and S4 Tables show genotype distributions for women and men).

### Identification of cooperative strategies

We classified each subject’s cooperative strategy into four types using the following classification algorithm. First, subjects whose maximum contribution in the contingent contribution table was below or equal to 20% of the endowment (4 tokens) were considered as free riders (FR). For strategies that did not enter the FR category, we proceeded as follows; for each strategy we ran two simultaneous Spearman rank correlations between the subject’s contingent contribution and others’ hypothetical average contribution. Initially, the first correlation considered the first three entries in the contingent contribution table (when others’ hypothetical average contribution was 0, 1, and 2) and the second correlation considered the rest of the entries in the contingent contribution table (when others’ hypothetical average contribution was 3, 4, …, 20). We repeated this procedure for each strategy by including each entry sequentially in the first correlation and removing it from the second correlation until the first correlation considered the first 18 entries in the contingent contribution table (when others’ hypothetical average contribution was 0, 1, …, 17) and the second correlation considered the last three entries in the contingent contribution table (when others’ hypothetical average contribution was 18, 19, 20). Strategies were classified as hump-shaped (HS) if they showed at least one positive-to-negative change between the first and second correlation in the sign of their Spearman correlation coefficient at a 1% significance level. The remaining strategies were classified as conditional cooperators (CC) if they displayed a significantly positive Spearman coefficient (at a 1% significance level). Following Fischbacher et al. [5], we classified all the strategies that did not fall into FR, HS, or CC as others (OT). The OT category consists of strategies that presented miscellaneous patterns of contributions, including unconditional cooperation (three players) (see S3 Fig for individual OT strategies). We ran robustness checks with different FR classification criteria which considered subjects whose maximum contribution in the contingent contribution table was below or equal to 10% and 30% of the endowment.

### Statistical analysis

We ran all our analysis separately for each sex since previous studies suggest sex-specific associations [e.g. 27, 39]. We applied Bonferroni correction to account for multiple hypotheses testing in each set of analyses. Associations between genetic variants and cooperative strategies were tested using a Fisher exact test (α = 0.008 given six hypotheses). Additionally, to test the relationship between specific genotypes and cooperative strategies we ran a multinomial logistic regression model with bootstrapped standard errors for each variant. Then, we calculated the marginal effects of each genotype on the probability of a subject displaying a given cooperative strategy (α = 0.00125 given 40 hypotheses).

Following Mertins et al. [27], we also tested whether mean contingent contributions differed between genotypes under three cooperative scenarios. The “low contribution scenario” is the first seven entries in the contingent contribution table (i.e. when the mean hypothetical others’ contribution goes from 0 to 6 tokens), the “mid contribution scenario” is the next seven entries in the table (i.e. when the mean hypothetical others’ contribution goes from 7 to 13 tokens), and the “high contribution scenario” is the last seven entries in the table (i.e. when the mean hypothetical others’ contribution goes from 14 to 20 tokens). We ran Kruskal-Wallis rank tests to test significant differences in mean contribution between genotypes for each variant under the three scenarios (α = 0.003 given 18 hypotheses) ant to test whether uninformed contributions significantly differed between genotypes for each variant (α = 0.008 given six hypotheses). All analyses were run in R Studio v1.1.456 except multinomial logistic regressions which were run in Stata v.12.0. Data and code are available at https://github.com/ignacia-rivera/genetics_coop.

## Results

**Fig 1:**
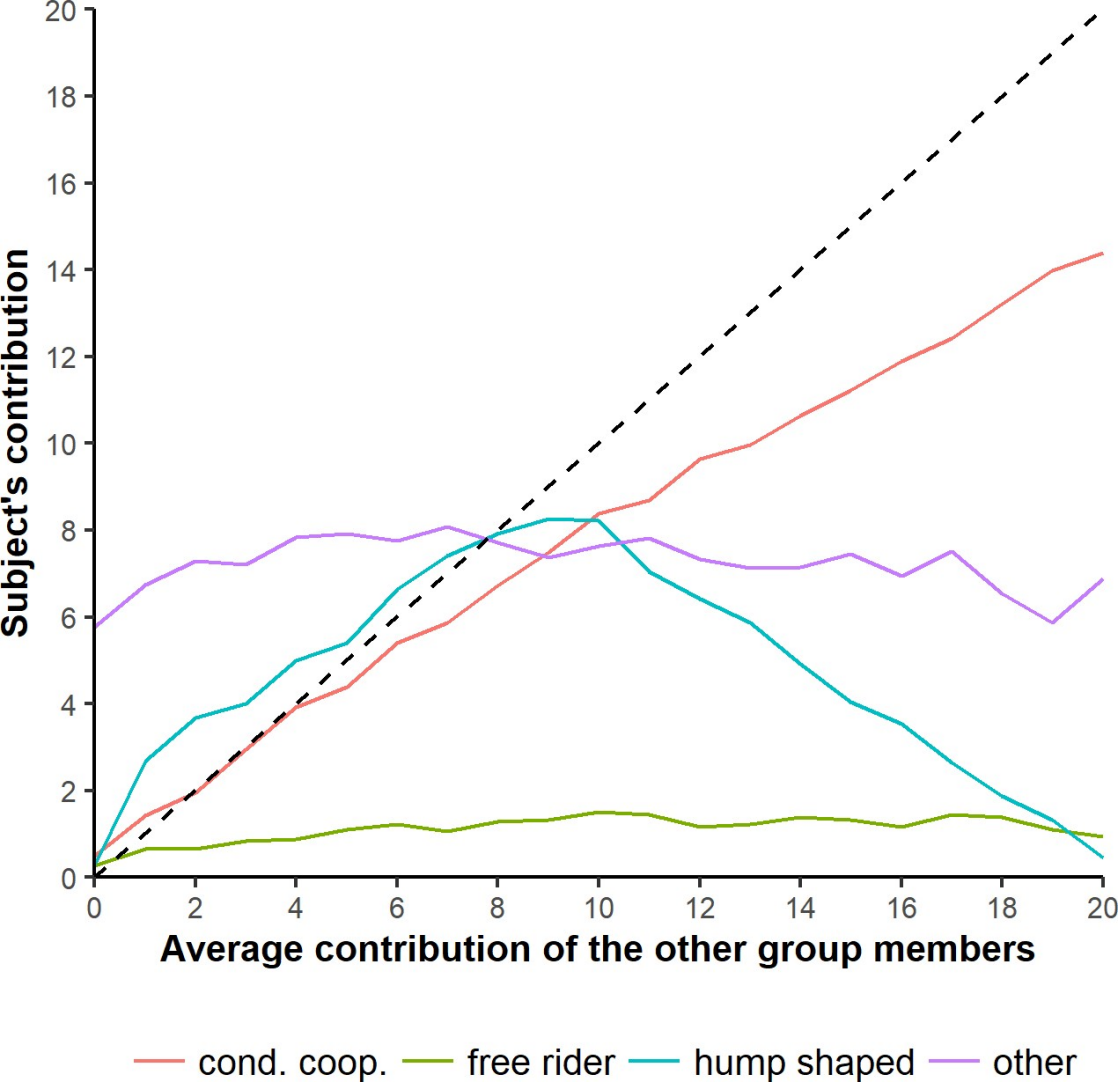
Average profile for each type of strategy. Average cooperative strategy for free riders (green), conditional cooperators (red), hump shaped (light blue) and others (purple). Dashed line represents the contribution profile of a perfect conditional cooperator.

The distribution of cooperative strategies is presented in Table 2. No significant difference was observed in the distribution of cooperative strategies of women and men (p = 0.545, two-sided Fisher test). The average profile for each type of strategy is shown in Fig 1. The average CC strategy deviates from the diagonal (perfect conditional cooperation) downwards displaying a bias towards selfishness.

**Table 2:** Percentage distribution of cooperative strategies.

Cooperative strategy	Percentage of women (%)	Percentage of men (%)
CC	44.86	54.32
HS	12.15	11.11
FR	9.35	9.88
OT	33.64	24.69

Percentage distribution of cooperative strategies for women (n = 107) and men (n = 81). Considering as a FR any strategy in which the maximum contingent contribution was equal or below 20% of the endowment.

**Fig 2:**
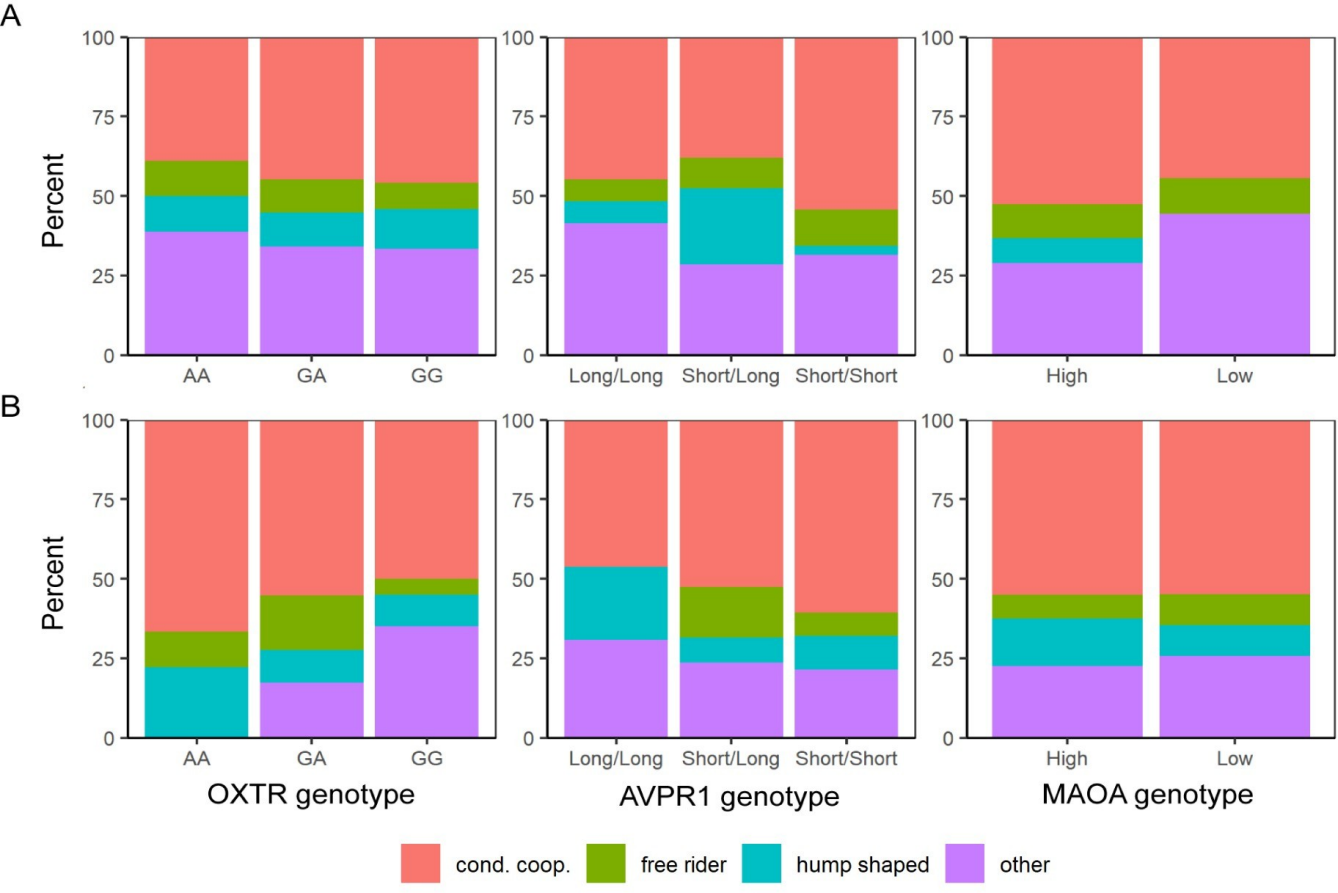
Distribution of cooperative strategies for each genotype. Percentage distribution of cooperative strategies, conditional cooperators (red), hump shaped (light blue), free riders (green) and others (purple) for AA (n = 18), GA (n = 38), and GG (n = 48) *OXTR* rs53576 genotypes; for Long/Long (n = 29), Short/Long (n = 42), and Short/Short (n = 35) *AVPR1a* RS3 genotypes; High (n = 9) and Low (n = 38) *MAOA* u-VNTR genotypes in women (panel A). Percentage distribution of cooperative strategies for AA (n = 9), GA (n = 29), and GG (n = 40) *OXTR* rs53576 genotypes; for Long/Long (n = 13), Short/Long (n = 38), and Short/Short (n = 28) *AVPR1a* RS3 genotypes; High (n = 31) and Low (n = 40) *MAOA* u-VNTR genotypes in men (panel B).

The distribution of cooperative strategies for each genotype is shown in Fig 2. No association between cooperative strategies and any of the variants was found, either for women or men (p ≥ 0.145, two-sided Fisher exact test). This result holds for classification criteria that use a cutoff of 10 and 30% of the endowment to characterize the FR strategy. Our regression analysis confirmed this result since no genotype was found to have a significant effect on the probability of a subject displaying a particular type of strategy after correcting for multiple hypotheses testing (p ≥ 0.01 with α = 0.00125, dy/dx from multinomial logistic regression, S5 and S6 Tables).

**Fig 3:**
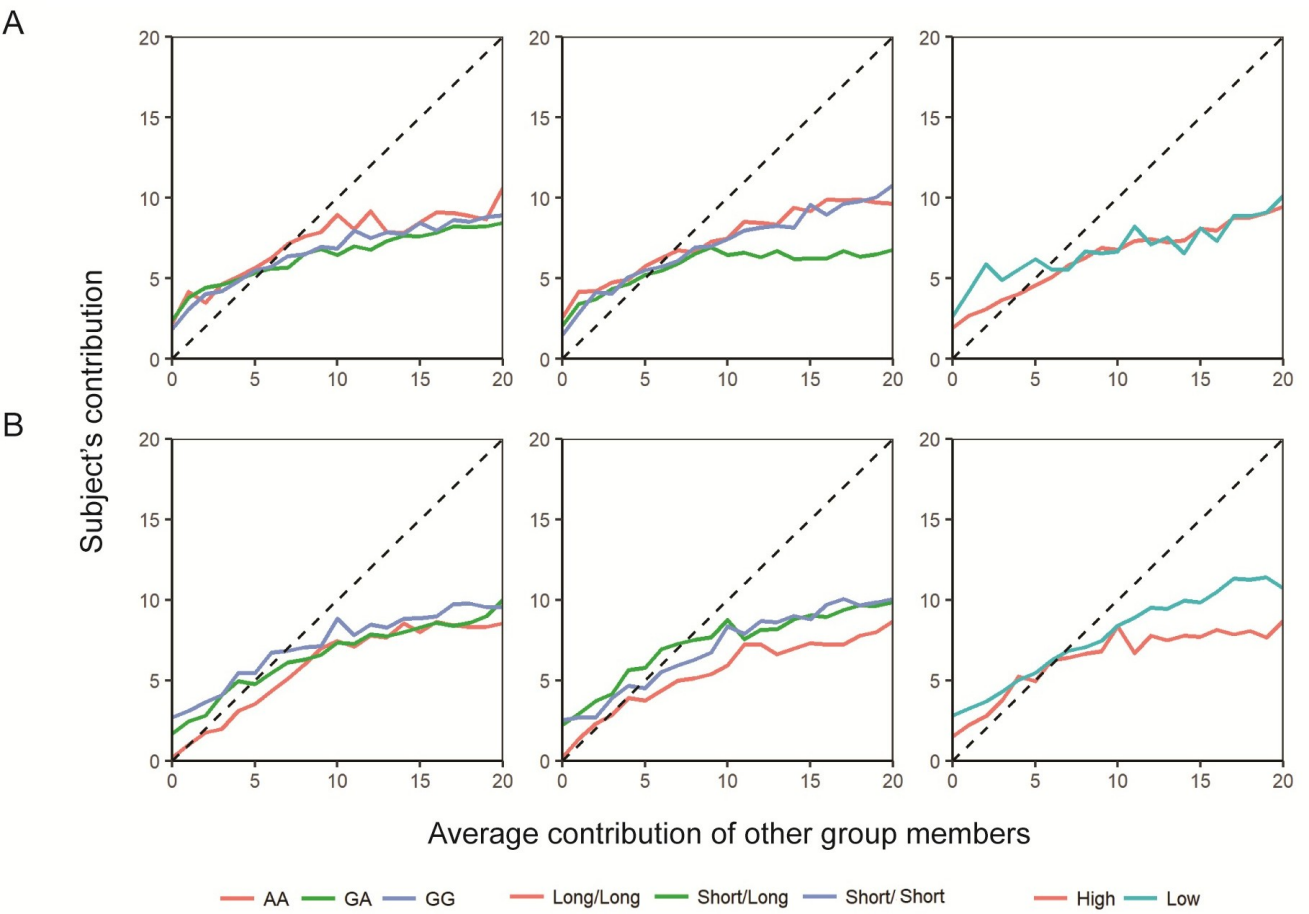
Average cooperative strategies per genotype. Average cooperative strategies per genotype for *OXTR* rs53576, *AVPR1a* RS3, and *MAOA* u-VNTR in women (panel A) and in men (panel B).

Some patterns could be distinguished between genotypes in their average strategy (Fig 3). For *OXTR* rs53756, AA women tended to contribute more than other genotypes when the average contribution of others was between around six and 14 tokens, while AA men seemed to contribute less than other genotypes when the average contribution of others was below 10 tokes. Women carrying Long and Short copies for *AVPR1a* RS3 reduced their levels of contribution once the average contribution of others reached approximately nine tokens relative to the homozygous types. Among men, Long/Long genotypes of *AVPR1a* RS3 presented strategies with generally lower contributions compared to the strategies of Short alleles carriers. MAOA-L women displayed strategies with higher contributions relative to MAOA-H women when the average contribution of others was less than approximately 6 tokens. In the case of men, MAOA-L showed higher contribution levels than MAOA-H when the average contribution of others was higher than 10 tokes. Despite these observed patterns, we found no statistically significant differences between genotypes regarding their mean contingent contribution under different cooperative scenarios (i.e. “high contribution”, “mid contribution” and “low contribution” scenarios) (p ≥ 0.025 with α = 0.003, Kruskal-Wallis rank test). No significant differences in uninformed contributions were found between genotypes for any of the variants neither for women nor men (p ≥ 0.18, Kruskal-Wallis).

## Discussion

Unlike most candidate gene studies that investigate associations with observable actions, we tested whether candidate variants are associated with underlying strategies. Our results showed no association between cooperative strategies and the three studied variants: *MAOA-*uVNTR, *OXTR* rs53576, and *AVPR1 RS3*. Therefore, our findings did not replicate previous results by Mertins et al. [27] for *MAOA*-uVNTR and did not match expected associations based on previous results linking *OXTR* rs53576, and *AVPR1* RS3 with sociality. This suggests that when cooperative phenotypes are measured more precisely as strategies—which exclude learning and expectations—associations with candidate genetic variants cannot be consistently replicated in small samples. This is in line with the consensus amongst geneticists that no single gene can explain a meaningful part of the variance observed in humans social traits [14, 15, 17–20, 46].

We characterize a cooperative phenotype as the subjects’ strategies in a PGG using the protocol by Fischbacher et al. [5]. The results from applying this method have been replicated in samples around the world, showing that the most prevalent strategy is CC [8]. To our knowledge, we report the first application of this protocol to elicit cooperative strategies in an admixed Latino sample. We replicated the main finding that the most frequent strategy is CC in both women and men, with frequencies that fall within the range of previous studies (from around 40% to 70% of trials) [8]. Typically, studies find that the second most frequent strategy is FR. In our sample, however, the second most frequent strategy was HS. Nonetheless, the frequencies we found for both HS and FR fall within the ranges observed in previous studies [8]. We found a high number of strategies that could not be categorized within the CC, FR, or HS categories (i.e. around 34% for women and 24% for men), which we classified as OT. The share of OT observed in our sample is high compared to what has been observed in most studies, but still within the ranges reported by other researchers [47]. The high number of OT in our sample relative to other studies can be due to differences in classification criteria as well as in populations. Overall, our behavioral results replicate broader strategy patterns found in previous studies and therefore provide a robust characterization of the cooperative inclinations defined in the experimental economics literature.

The strategy method purposely minimizes the effect that others have on individual decisions to elicit a controlled measure of cooperative preferences. Yet, cooperative interactions also involve social cognitive processes such as emotion recognition [48, 49], empathy and theory of mind [50], social communication [51], and social reward seeking [52]. All of those are excluded from our measurement of cooperation and could be influenced by the genetic variants studied here. Indeed, a study by [29] suggests that variation in *MAOA-uVNTR* correlates with differences in expectations about others’ behaviors, and variations in *OXTR* have been associated with empathy [53] and social reward [12, 54]. Furthermore, it has been suggested that empathy and perspective taking mediate the effects of *OXTR* on prosocial behavior [55]. This highlights the importance of disentangling the multiple cognitive phenomena involved in complex behaviors such as cooperation when aiming to link them to variation in candidate genes.

At least, three reasons can explain the lack of replicability and mismatch with previous related observations. First, our admixed Latino population differs, both genetically and environmentally, from the Caucasian and Asian populations commonly studied in similar candidate gene studies (see **Table 1**). Variability in associations between populations can arise due to differences in gene-environment interactions [56]. Indeed, it has been suggested that social behaviors are influenced by culture which can mask genetic influences differentially across populations [57]. Different patterns of linkage disequilibrium can also explain differences in gene-trait associations across populations [58].

A second possible reason why we did not find the associations suggested by previous studies is that previous studies may have misrepresented the association between these candidate variants and cooperative traits. Despite having selected our candidate variants based on an exhaustive literature review, new evidence has come to question common findings in this body of research. In particular, there are serious methodological concerns about the validity of several observations linking oxytocin with trust, which is one of the most studied associations in social neuroscience [59–62]. For instance, the association between exogenous intranasal oxytocin and higher levels of trust [54, 63] and the correlation between trust and oxytocin plasma levels [64] has been poorly replicated [60, 65]. Lack of robust results linking trust with *OXTR* has also been evidenced in candidate gene studies. For example, while [66] reported a significant association between *OXTR* rs53576 and investments in a trust game, [67] reported no association in a larger sample (N = 684). Moreover, many results of candidate gene studies are thought to be false positives since most of them do not account for family-wise error [46]. Indeed, if we had not corrected for multiple hypotheses testing, we would have observed significant misleading associations. All this demonstrates the susceptibility of candidate gene studies to fall into biases by following genetic variants overrepresented in the literature and underscores the value of publishing null results.

The third explanation for the lack of associations observed in our study is insufficient statistical power. In theory, more refined measurements of social phenotypes should increase a study’s capacity to detect associations between genes and social traits. Nonetheless, our results suggest that refining the characterization of cooperative phenotypes is not enough to overcome the problem of inherently low statistical power of candidate gene studies. This detection problem is fundamentally due to the small effects that single genes have on complex social behaviors, such that they would require massive sample sizes to be detected. Consequently, geneticists seriously question the value of candidate gene studies to understand the underlying genetics of complex social behaviors [14, 17–19].

Successful candidate gene studies would require sufficiently large samples and candidate variants that have a credible high prior probability of being associated with the trait of interest [19, 46]. Therefore, there is still a need to better understand the links between genotype and cooperation using approaches with higher statistical power before implementing promising candidate genes studies. Genome-wide association studies (GWAS) ―which simultaneously explore thousands of variants while accounting for family-wise error in a hypothesis generating manner ― have a lot to offer in terms of pointing to potentially relevant genetic variants [68–72]. Nonetheless, GWAS that directly explore cooperation are still lacking and those that have looked into similar prosocial constructs have not found significant associations [14, 73]. Studies involving neuroimaging and neurotransmitter measurements are also promising to identify neurobiological pathways involved in cooperative decision making [38, 74]. These studies can further point to promising candidate genes by narrowing down the neural structures, molecules, and networks involved in cooperative decisions.

We explored whether refining the measurement of cooperative phenotypes as strategies rather than actions increases the capacity of a candidate gene study to replicate associations between candidate variants and cooperation in a small sample. Our results suggest that this approach alone cannot solve the inherent statistical power problem of this type of studies. Nonetheless, the refinement of cognitive constructs in GWAS and their proper measurement is still a promising approach to improve our ability to detect genes associated with complex behaviors. Better measurements can be informed by novel designs developed by behavioral scientists that allow unpacking decisions involving multiple cognitive phenomena like the strategy method implemented in our study.

## Supporting information

S1 Text Game instructions. Instructions of the game presented to the subjects in Spanish.
(DOCX)

S1 Fig Screen 1. Screen to collect decisions for the “uninformed player” role (text in Spanish).
(DOCX)

S2 Fig Screen 2. Screen to collect decisions for the “informed player” role (text in Spanish).
(DOCX)

S3 Fig Individual schedules for strategies categorized as those of others. Each plot shows the strategies of five individuals except the last one which shows six.
(DOCX)

S1 Table Frequency of *MAOA* u-VNTR alleles. (DOCX)

S2 Table Frequency and classification of *AVPR1a* RS3 alleles based on length (number of base pairs). (DOCX)

S3 Table Frequency of the genotypes for each variant in women. (DOCX)

S4 Table Frequency of the genotypes for each variant in men. (DOCX)

S5 Table Marginal effects of each genotype on each cooperative strategy in women. Obtained from a multinomial logistic regression model for each genetic variant that uses cooperative strategy as a dependent variable and genotypes as independent variables. For *OXTR* rs53567 AA is the baseline genotype (n = 104), for *AVPR1* RS3 Long/Long is the baseline genotype (n = 106), and for *MAOA* u-VNTR the Low expression is the baseline genotype (n = 47).
(DOCX)

S6 Table Marginal effects of each genotype on each cooperative strategy in men. Obtained from a multinomial logistic regression model for each genetic variant that used cooperative strategy as a dependent variable and genotypes as independent variables. For *OXTR* rs53567 AA is the baseline genotype (n = 78), for *AVPR1* RS3 Long/Long is the baseline genotype (n = 79), and for *MAOA* u-VNTR the Low expression is the baseline genotype (n = 71).
(DOCX)

S1 Data (CSV)
