# Supplementary material for: No association between genetic variants in MAOA, OXTR, and AVPR1a and cooperative strategies
Source: PLoS One. 2020 Dec 23;15(12):e0244189. doi: 10.1371/journal.pone.0244189 (PMC7757875; doi:10.1371/journal.pone.0244189)
Supplement: S1 Text — Instructions of the game presented to the subjects in Spanish. (DOCX) [file pone.0244189.s001.docx]

**S1 Text. Game instructions**

The public good game is played in groups of four. Groups will be formed randomly by the software with people in this room. All the interactions will be conducted through the computers and you will never know the identity of the three people you played with.

At the beginning of the game each subject will receive 20 tokens. Next, subjects must decide individually how many tokens to keep for themselves and how many to contribute to the public good. Once every player has decided on their contributions, the number of tokens in the public good gets doubled. Next, the public good is divided in equal parts amongst the four players (fractions of tokens will be rounded to the integer). Thus, for every token that a player contributes to the public good, that player receives back half a token and the other three players receive half a token each.

The end of the game is given by the reparation of the public good. At that moment, tokens are transformed into their monetary value. Each token is valued in $250 CLP. Therefore, earnings can range from $2.500 to $12.500 CLP in the game depending on how many tokens they and the other players in the group decide to contribute.

Decisions in the game will be recorded anonymously. Neither the other participants nor the researchers will be able to associate decisions to personal identities. While playing the game, you must remain silent. Earnings will be paid in private in a separate room at the end of the session.

The following table shows an example of the game:

Imagine 36 tokens were contributed to the public good. These tokens will be doubled up to 72 tokens. Then, they will be equally distributed amongst the four players in the group. Thus, each will receive 18 tokens plus the tokens each decided to keep for themselves.

| Player | Tokens contributed to the public good | Tokens kept | Tokens from the public good | Total tokens | Earnings (CLP) |
| --- | --- | --- | --- | --- | --- |
| A | 0 | 20 | 18 | 38 | 9.500 |
| B | 19 | 1 | 18 | 19 | 4.750 |
| C | 6 | 14 | 18 | 32 | 8.000 |
| D | 11 | 9 | 18 | 27 | 6.750 |

The payoffs will be computed as follows. Three players will be chosen randomly by the software to play as “uninformed players”, they will play without knowing the contributions of the other players in the group. The fourth player will play as an “informed player”, who plays knowing the average number of tokens that the other players in the group contributed to the public good. Thus, the fourth player can condition her/his contribution on the average contribution of other players in the group.

The decision of the “informed player” is a plan rather than a single number. For example, the plan of an “informed player” could be the following:

If the other players in my group contribute on average 0 tokens, I will contribute 0 tokens,

if the other players in my group contribute on average 2 tokens, I will contribute 15 tokens,

if the other player in my group contribute on average 4 tokens, I will contribute 7 tokens,

and so on…

Let us imagine that the “uninformed players” contribute 1, 6 and 5 tokens to the public good, respectively. The average contribution is 4 tokens. Based on the previous plan, the “informed player” will contribute 7 tokens. Thus, the total contribution to the public good is 1 + 6 + 5 +7 = 19. The public good gets doubled up to 38 tokens which are divided in equal parts amongst the four players. Therefore, each player receives 9.5 tokens form the public good, which rounded to the integer are 10 tokens. Since the “informed player” kept 13 tokens for her/himself, her/his total number of tokens is 13 + 10 = 23 and her/his monetary earnings are 23 × $250 CLP = $5.750 CLP.

When deciding, no player will know whether they will play as the “uninformed player” or as the “informed player”. Thus, each player will provide both types of decisions and then the software will randomly decide in which role she/he will play. To provide both types of decisions you will be asked to answer two questions:

1. How many tokens will you contribute as an “uniformed player? (**S2 Figure** was presented to the subjects)
2. What is your plan if you contribute as an “informed player”? (**S3 Figure** was presented to the subjects)
