# Supplementary figures and images for: No association between genetic variants in MAOA, OXTR, and AVPR1a and cooperative strategies
Source: PLoS One. 2020 Dec 23;15(12):e0244189. doi: 10.1371/journal.pone.0244189 (PMC7757875; doi:10.1371/journal.pone.0244189)

**S2 Fig. Screen 1.** Screen to collect decisions as “uninformed players” (text in Spanish)**.**


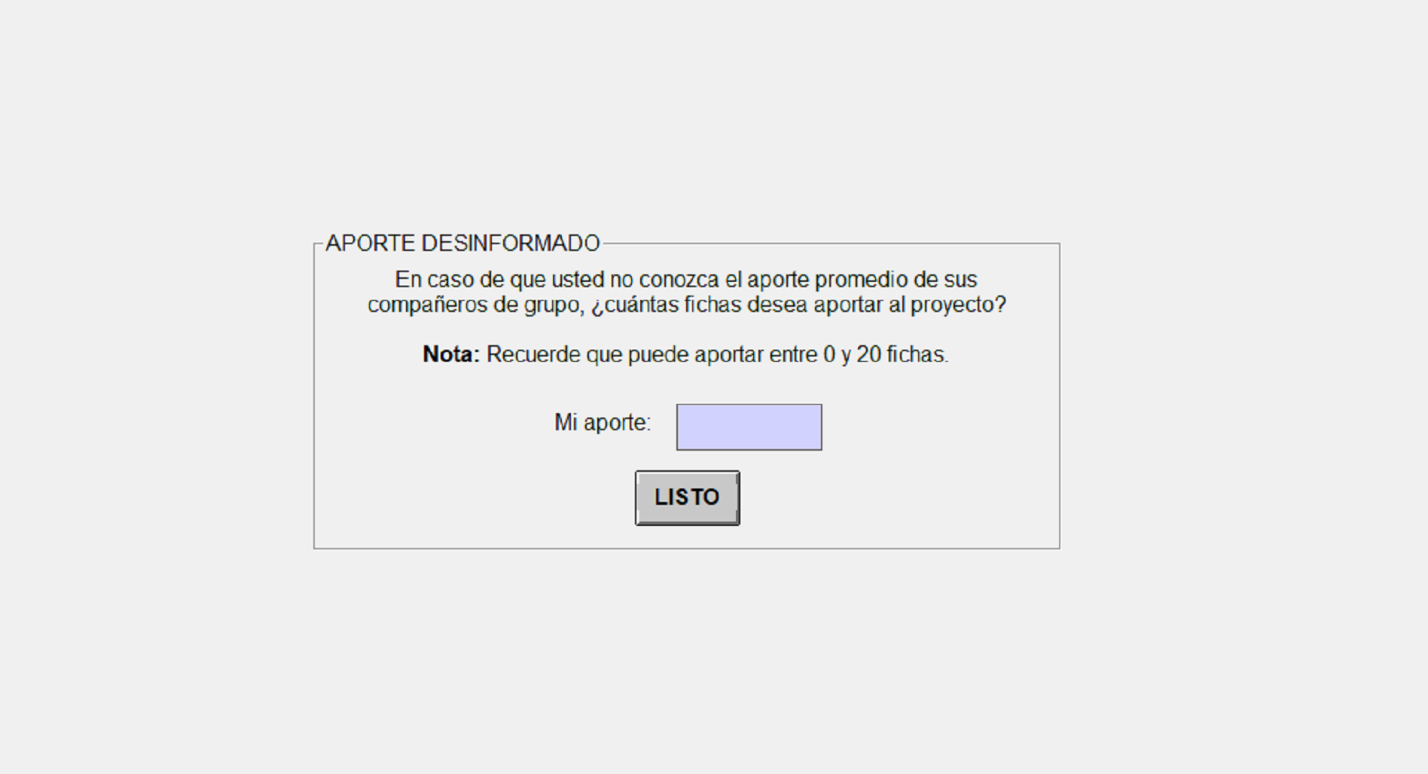

Supplement: S1 Fig — Screen to collect decisions for the “uninformed player” role (text in Spanish). (DOCX) [file pone.0244189.s002.docx]

**S3 Fig. Screen 2.** Screen to collect decisions for the “informed player” role (text in Spanish)**.**


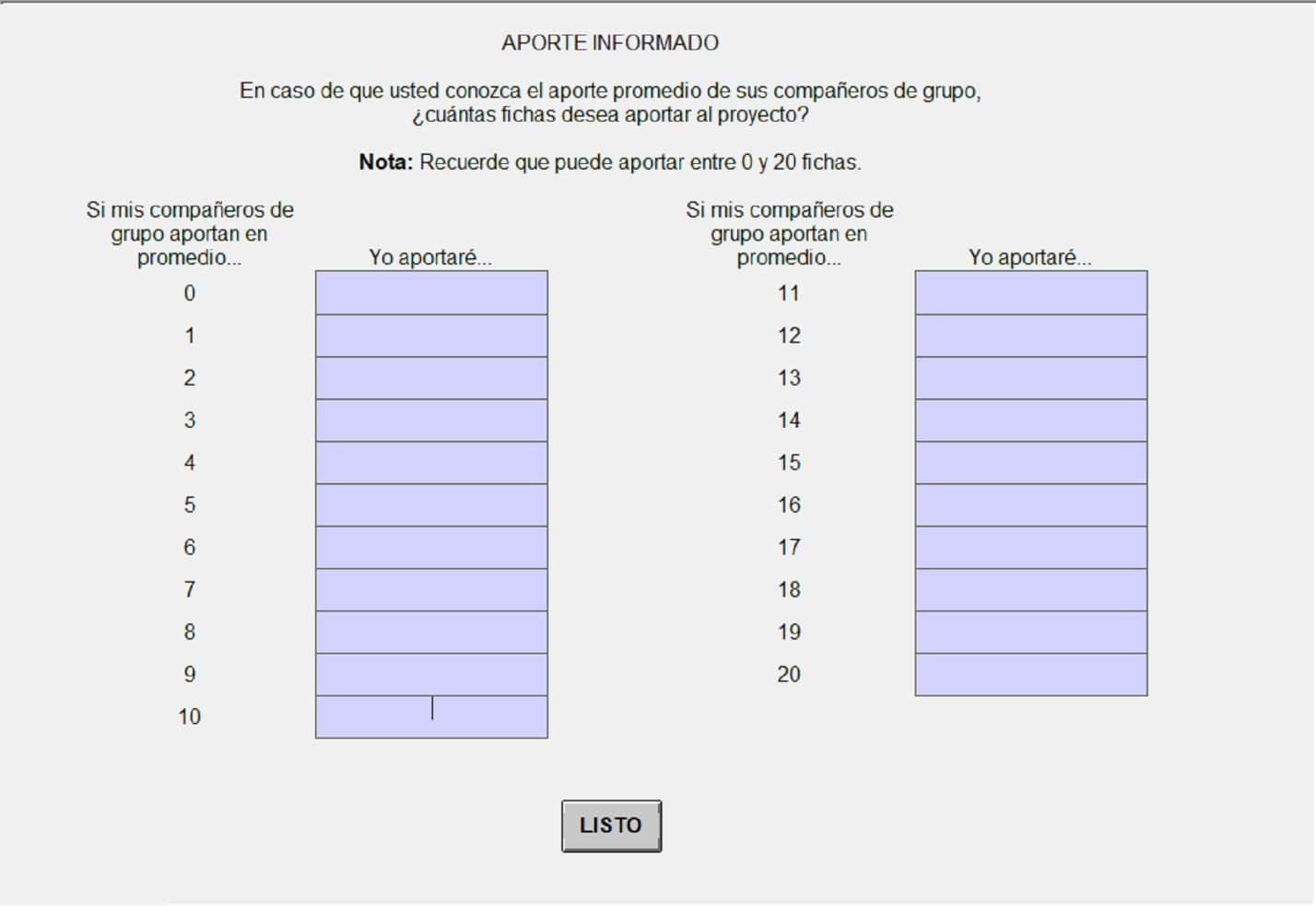

Supplement: S2 Fig — Screen to collect decisions for the “informed player” role (text in Spanish). (DOCX) [file pone.0244189.s003.docx]
