# Supplementary material for: No association between genetic variants in MAOA, OXTR, and AVPR1a and cooperative strategies
Source: PLoS One. 2020 Dec 23;15(12):e0244189. doi: 10.1371/journal.pone.0244189 (PMC7757875; doi:10.1371/journal.pone.0244189)
Supplement: S3 Fig — Each plot shows the strategies of five individuals except the last one which shows six. (DOCX) [file pone.0244189.s004.docx]

**S8 Fig. Individual schedules for strategies categorized as those of others**. Each plot shows the strategies of five individuals, except the last one, which shows six.

**
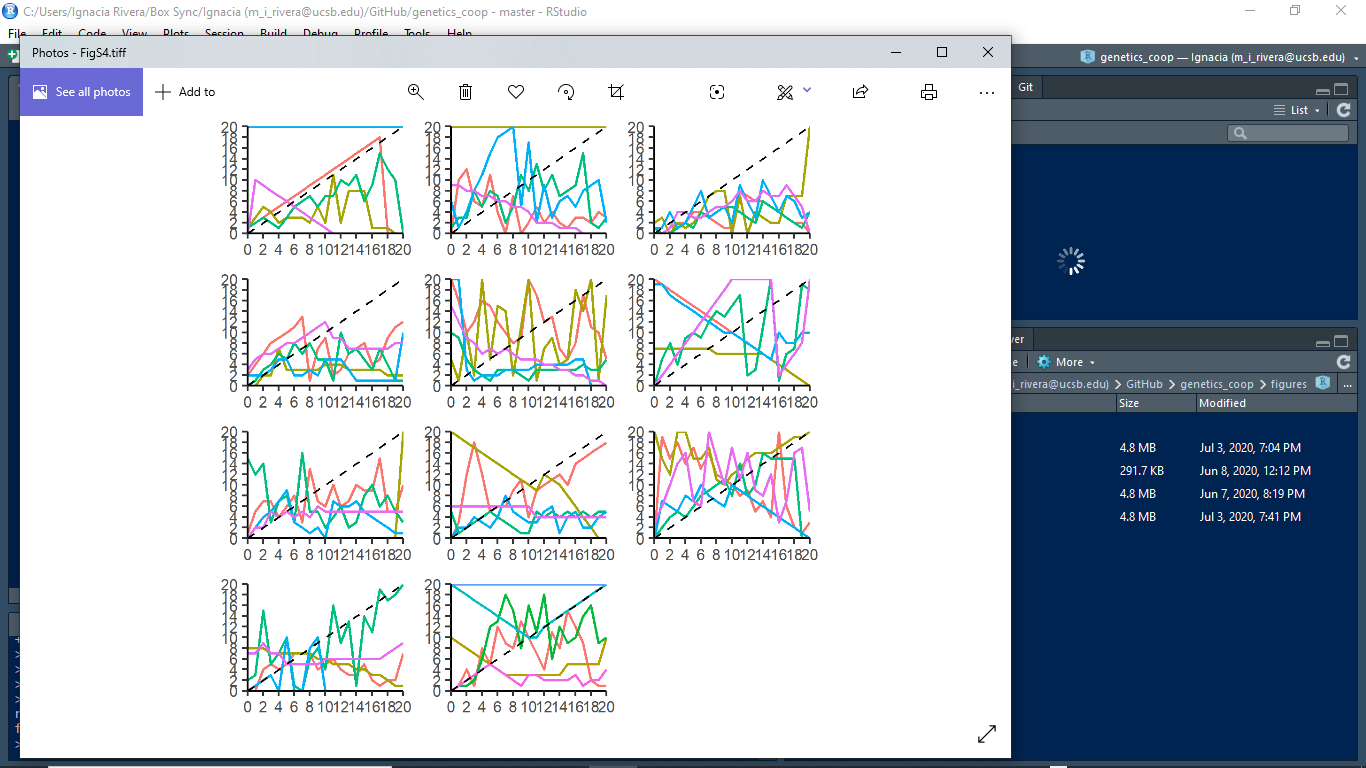
**
