# Supplementary material for: No association between genetic variants in MAOA, OXTR, and AVPR1a and cooperative strategies
Source: PLoS One. 2020 Dec 23;15(12):e0244189. doi: 10.1371/journal.pone.0244189 (PMC7757875; doi:10.1371/journal.pone.0244189)
Supplement: S1 Table — (DOCX) [file pone.0244189.s005.docx]

**S1 Table**. **Frequency of *MAOA* u-VNTR alleles**

| **Allele (number of repeats)** | **Frequency** |
| --- | --- |
| 3.5 | 92 |
| 4.5 | 158 |
| 5.5 | 3 |
| 6.5 | 1 |
