# Supplementary material for: No association between genetic variants in MAOA, OXTR, and AVPR1a and cooperative strategies
Source: PLoS One. 2020 Dec 23;15(12):e0244189. doi: 10.1371/journal.pone.0244189 (PMC7757875; doi:10.1371/journal.pone.0244189)
Supplement: S2 Table — (DOCX) [file pone.0244189.s006.docx]

**S2 Table. The frequency and classification of *AVPR1a* RS3 alleles based on length (number of base pairs)**

| **Allele (bp)** | **Frequency** | **Length classification** |
| --- | --- | --- |
| 324 | 5 | Short |
| 325 | 2 |  |
| 326 | 1 |  |
| 331 | 1 |  |
| 333 | 1 |  |
| 334 | 1 |  |
| 335 | 18 |  |
| 337 | 23 |  |
| 338 | 2 |  |
| 339 | 80 |  |
| 341 | 72 |  |
| 342 | 1 | Long |
| 343 | 38 |  |
| 344 | 1 |  |
| 345 | 58 |  |
| 346 | 1 |  |
| 347 | 21 |  |
| 348 | 5 |  |
| 349 | 2 |  |
| 351 | 18 |  |
| 352 | 3 |  |
| 353 | 10 |  |
| 354 | 5 |  |
| 356 | 1 |  |
