# Supplementary material for: No association between genetic variants in MAOA, OXTR, and AVPR1a and cooperative strategies
Source: PLoS One. 2020 Dec 23;15(12):e0244189. doi: 10.1371/journal.pone.0244189 (PMC7757875; doi:10.1371/journal.pone.0244189)
Supplement: S3 Table — (DOCX) [file pone.0244189.s007.docx]

**S3 Table. Frequency of the genotypes for each genetic variant in women**

| ***OXTR* rs53576** | **n** | ***AVPR1* RS3** | **n** | ***MAOA* u-VNTR** | **n** |
| --- | --- | --- | --- | --- | --- |
| GG | 48 | Long/Long | 29 | 3.5/3.5 or Low | 9 |
| GA | 38 | Long/Short | 42 | 3.5/4.5 * | 41 |
| AA | 18 | Short/Short | 35 | 4.5/4.5 or High | 38 |
| Not amplified | 3 | Not amplified | 1 | Not amplified or excluded** | 19 |

* The genotype 3.5/4.5 repeats were excluded from the association analysis for *MAOA* u-VNTR since it is not possible to know which one is being expressed in women.

** Given the low frequency of the 5.5 and 6.5 repeats alleles in our sample, we excluded their carriers from the association analysis for *MAOA* u-VNTR.
