# Supplementary material for: No association between genetic variants in MAOA, OXTR, and AVPR1a and cooperative strategies
Source: PLoS One. 2020 Dec 23;15(12):e0244189. doi: 10.1371/journal.pone.0244189 (PMC7757875; doi:10.1371/journal.pone.0244189)
Supplement: S4 Table — (DOCX) [file pone.0244189.s008.docx]

**S4 Table. Frequency of the genotypes for each variant in men.**

| ***OXTR* rs53756** | **n** | ***AVPR1* RS3** | **n** | ***MAOA* u-VNTR** | **n** |
| --- | --- | --- | --- | --- | --- |
| GG | 40 | Long/Long | 13 | 3.5 or Low | 31 |
| GA | 29 | Long/Short | 38 | 4.5 or High | 40 |
| AA | 9 | Short/Short | 28 | - | - |
| Not amplified | 3 | Not amplified | 2 | Not amplified or excluded * | 10 |

* Given the low frequencies of the 5.5 and 6.5 repeats alleles in our sample, we excluded their carriers from the association analysis for *MAOA* u-VNTR.
