# Supplementary material for: No association between genetic variants in MAOA, OXTR, and AVPR1a and cooperative strategies
Source: PLoS One. 2020 Dec 23;15(12):e0244189. doi: 10.1371/journal.pone.0244189 (PMC7757875; doi:10.1371/journal.pone.0244189)
Supplement: S5 Table — Obtained from a multinomial logistic regression model for each genetic variant that uses cooperative strategy as a dependent variable and genotypes as independent variables. For OXTR rs53567 AA is the baseline genotype (n = 104), for AVPR1 RS3 Long/Long is the baseline genotype (n = 106), and for MAOA u-VNTR the Low expression is the baseline genotype (n = 47). (DOCX) [file pone.0244189.s009.docx]

**S5 Table. Marginal effects of each genotype on each cooperative strategy in women.** Obtained from a multinomial logistic regression model for each variant that uses cooperative strategy as a dependent variable and genotypes as independent variables. For *OXTR* rs53567 AA is the baseline genotype (n=104), for *AVPR1* RS3 Long/Long is the baseline genotype (n=106), and for *MAOA* u-VNTR the Low expression is the baseline genotype (n=47).

| **Genetic variant** | **Cooperative strategy** | **Genotype** | **Marginal effect** | **p-value** |
| --- | --- | --- | --- | --- |
| *OXTR* rs53576 | CC | AG | 0.06 (*0.42* ) | 0.89 |
|  |  | GG | 0.07 (*0.38*) | 0.85 |
|  | HS | AG | -0.01 (*0.49*) | 0.99 |
|  |  | GG | 0.01 (*0.44*) | 0.98 |
|  | FR | AG | -0.01 (*0.55*) | 0.99 |
|  |  | GG | -0.03 (*0.52*) | 0.96 |
|  | OT | AG | -0.05 (0*.39*) | 0.91 |
|  |  | GG | -0.06 (0*.39*) | 0.87 |
| *AVPR1* RS3 | CC | Short/Long | -0.07 (*0.24*) | 0.78 |
|  |  | Short/Short | 0.09 (*0.30*) | 0.75 |
|  | HS | Short/Long | 0.17 (*0.32*) | 0.60 |
|  |  | Short/Short | -0.04 (*0.39*) | 0.92 |
|  | FR | Short/Long | 0.03 (*0.31*) | 0.93 |
|  |  | Short/Short | 0.05 (*0.41*) | 0.91 |
|  | OT | Short/Long | -0.13 (*0.23*) | 0.58 |
|  |  | Short/Short | -0.10 (*0.24*) | 0.679 |
| *MAOA* u-VNTR | CC | High | 0.08 (*0.99*) | 0.94 |
|  |  |  |  |  |
|  | HS | High | 0.08 (*0.04*) | 0.07 |
|  |  |  |  |  |
|  | FR | High | -0.01 (*0.85*) | 0.99 |
|  |  |  |  |  |
|  | OT | High | -0.16 (*0.77*) | 0.84 |
|  |  |  |  |  |
