# Supplementary material for: No association between genetic variants in MAOA, OXTR, and AVPR1a and cooperative strategies
Source: PLoS One. 2020 Dec 23;15(12):e0244189. doi: 10.1371/journal.pone.0244189 (PMC7757875; doi:10.1371/journal.pone.0244189)
Supplement: S6 Table — Obtained from a multinomial logistic regression model for each genetic variant that used cooperative strategy as a dependent variable and genotypes as independent variables. For OXTR rs53567 AA is the baseline genotype (n = 78), for AVPR1 RS3 Long/Long is the baseline genotype (n = 79), and for MAOA u-VNTR the Low expression is the baseline genotype (n = 71). (DOCX) [file pone.0244189.s010.docx]

**S6 Table. Marginal effects of each genotype on each cooperative strategy in men.** Obtained from a multinomial logistic regression model for each variant that used cooperative strategy as a dependent variable and genotypes as independent variables. For *OXTR* rs53567 AA is the baseline genotype (n=78), for *AVPR1* RS3 Long/Long is the baseline genotype (n=79), and for *MAOA* u-VNTR the Low expression is the baseline genotype (n=71).

| **Genetic variant** | **Cooperative strategy** | **Genotype** | **Marginal effect** | **p-value** |
| --- | --- | --- | --- | --- |
| *OXTR* rs53576 | CC | AG | -0.12 (*1.15*) | 0.92 |
|  |  | GG | -0.17 (*1.11*) | 0.88 |
|  | HS | AG | -0.12 (*1.15*) | 0.92 |
|  |  | GG | -0.12 (*1.13*) | 0.91 |
|  | FR | AG | 0.06 (*0.79*) | 0.94 |
|  |  | GG | -0.06 (*0.85*) | 0.94 |
|  | OT | AG | 0.17 (*0.09*) | 0.05 |
|  |  | GG | 0.35 (*0.14*) | 0.01 |
| *AVPR1* RS3 | CC | Short/Long | 0.07 (*0.39*) | 0.87 |
|  |  | Short/Short | 0.15 (*0.55*) | 0.79 |
|  | HS | Short/Long | -0.15 (*0.59*) | 0.80 |
|  |  | Short/Short | -0.12 (*0.68*) | 0.86 |
|  | FR | Short/Long | 0.16 (*0.08*) | 0.04 |
|  |  | Short/Short | 0.07 (*0.36*) | 0.84 |
|  | OT | Short/Long | -0.07 (*0.27*) | 0.79 |
|  |  | Short/Short | -0.09 (*0.34*) | 0.78 |
| *MAOA* u-VNTR | CC | High | 0.00 (0.28) | 0.99 |
|  |  |  |  |  |
|  | HS | High | 0.05 (0.21) | 0.80 |
|  |  |  |  |  |
|  | FR | High | -0.02 (0.37) | 0.95 |
|  |  |  |  |  |
|  | OT | High | -0.03 (0.16) | 0.83 |
|  |  |  |  |  |
